# Seasonal Patterns of Soil Respiration and Related Soil Biochemical Properties under Nitrogen Addition in Winter Wheat Field

**DOI:** 10.1371/journal.pone.0144115

**Published:** 2015-12-02

**Authors:** Guopeng Liang, Albert A. Houssou, Huijun Wu, Dianxiong Cai, Xueping Wu, Lili Gao, Jing Li, Bisheng Wang, Shengping Li

**Affiliations:** National Engineering Laboratory for Improving Quality of Arable Land, Institute of Agricultural Resources and Regional Planning, Chinese Academy of Agricultural Sciences, Beijing, PR China; Chinese Academy of Sciences, CHINA

## Abstract

Understanding the changes of soil respiration under increasing N fertilizer in cropland ecosystems is crucial to accurately predicting global warming. This study explored seasonal variations of soil respiration and its controlling biochemical properties under a gradient of Nitrogen addition during two consecutive winter wheat growing seasons (2013–2015). N was applied at four different levels: 0, 120, 180 and 240 kg N ha^-1^ year^-1^ (denoted as N0, N12, N18 and N24, respectively). Soil respiration exhibited significant seasonal variation and was significantly affected by soil temperature with Q_10_ ranging from 2.04 to 2.46 and from 1.49 to 1.53 during 2013–2014 and 2014–2015 winter wheat growing season, respectively. Soil moisture had no significant effect on soil respiration during 2013–2014 winter wheat growing season but showed a significant and negative correlation with soil respiration during 2014–2015 winter wheat growing season. Soil respiration under N24 treatment was significantly higher than N0 treatment. Averaged over the two growing seasons, N12, N18 and N24 significantly increased soil respiration by 13.4, 16.4 and 25.4% compared with N0, respectively. N addition also significantly increased easily extractable glomalin-related soil protein (EEG), soil organic carbon (SOC), total N, ammonium N and nitrate N contents. In addition, soil respiration was significantly and positively correlated with β-glucosidase activity, EEG, SOC, total N, ammonium N and nitrate N contents. The results indicated that high N fertilization improved soil chemical properties, but significantly increased soil respiration.

## Introduction

Soil respiration has a great impact on atmospheric CO_2_, with about 80.4Pg C being emitted to the atmosphere every year [1]. Cropland ecosystem occupies more than 10% of the land globally, hosting 170 Pg of soil organic C [2]. Changes in soil respiration that result in net changes of soil carbon stocks may result in important positive or negative feedbacks to the climate system [3].

Two types of factors can affect soil respiration: abiotic factors, such as soil moisture, temperature, nitrogen availability, organic matter, etc., and biotic factors, which include microbial and enzymatic activities, root biomass [4]. Nitrogen addition can stimulate respiration variation by regulating plants growth, and influencing microbial activities [5–7] which are directly associated with CO_2_ production. Even though some studies suggest N fertilization reduces soil respiration [8–9], a positive correlation between soil respiration and N addition is also found [10]. Understanding soil respiration responses to N is particularly important in view of the fact that, over the past century, anthropogenic activities have doubled the rate of N inputs to most terrestrial ecosystems, and in many areas these rates are still increasing [11].

Soil temperature and moisture are considered as the dominant factors of soil respiration. The exponential relationship between soil respiration and soil temperature was reported by many studies [12–13]. In both arid and semi-arid ecosystems, low soil temperature inhibits soil respiration, high soil temperature accelerates it [14–16]. A significant and positive correlation between soil respiration and soil surface temperature was observed in a wheat-corn rotation cropland of the North China Plain [17]. However, effects of soil moisture on soil respiration are not always consistent. Both low and high soil moisture reduce soil respiration by inhibiting respiration substrate availability [18] and blocking CO_2_ transport [19], respectively. However, positive correlation between soil respiration and moisture was found by many previous studies [20–21].

Glomalin, which is produced by arbuscular mycorrhizal fungi (AMF), contributes to the preservation of organic carbon in the soil [22]. Some authors suggested that glomalin can produce a positive influence on SOC and available N [23–24]. However, few studies so far have been done to certificate whether glomalin can significantly affect soil respiration [25], further studies are necessary to determine the relationship between glomalin and soil respiration. Bradford method might be useful in measuring glomalin pools when organic matter concentrations were low or under controlled experimental conditions [26]. SOC and nutrients (total N, available P and available K) at our study site are generally low. The method was also used to measure glomalin content by many recent studies [25, 27]. Some soil enzymes such as β-glucosidase and Cellobiohydrolase are specific for the degradation of soil organic matter [28–29], while others such as N-acetyl-β-D-glucosaminidase and urease are involved in organic nitrogen mineralization [30–31]. Some studies showed that high application of N fertilizer can increase the activities of enzymes related to C acquisition, but decrease the activities of some enzymes involved in organic nitrogen N acquisition [32–33]. Gispert et al. (2013) reported that there was a significant positive correlation between β-glucosidase activity and soil respiration [25].

The effects of these factors on soil respiration vary across ecosystems in different regions, and quantitatively describing the impacts of main biotic and abiotic factors on soil respiration is necessary to accurately estimate soil respiration. In the North China Plain, arable soils are intensively cultivated with high inputs of N fertilizer to get good yield, which is not beneficial to agricultural sustainable development. However, there is very limited information on the responses of soil respiration to N fertilization in winter wheat cropland, especially on how N addition affects soil respiration by influencing glomalin and soil enzyme activities. We conducted a controlled experiment to answer the following questions: (i) what is the seasonal variation of soil respiration in winter wheat growing season? and (ii) could N addition significantly affect soil respiration? and (iii) are there significant correlations between enzymatic activities, glomalin content and soil respiration?

## Materials and Methods

### Ethics Statement

The study was conducted at Langfang Agricultural Station of Chinese Academy of Agricultural Sciences, Hebei Province, China. Permission was obtained from the Station administration to allow sampling. No rare or endangered wild animals or plants were collected in this experiment. Furthermore, this study did not use wild animals or plants as research objects and did not threaten the environment system.

### Site description

The experiment was established in 2009 at Langfang Agricultural Station of Chinese Academy of Agricultural Sciences (39°36′N, 116°36′E, 18 masl), Hebei Province, China. The climate is temperate continental monsoon and average annual temperature is 11.9°C. The mean annual precipitation is 550 mm and 80% of falls occurs between June and September. The soil texture is silt loam according to the FAO soil classification system [34], and soil physical-chemical properties before experiment are 6.38 g kg^-1^ soil organic C, 0.85 g kg^-1^ total N, 12.75 mg kg^-1^ available P (Olsen method), 93.7 mg kg^-1^ available K (NH_4_AC extraction, atom absorb spectrophotometer (AAS) method), pH (H_2_O) 8.0.

### Experimental design and sampling

At the site, twelve 10.4m×6.4m plots were randomly selected and subjected to four nitrogen addition treatments: N0, N12, N18 and N24 (0, 120, 180 and 240 kg N ha^-1^ year^-1^, respectively). Nitrogen fertilizer was applied to the soil in the form of urea, 50% of N was applied as basal fertilizers just prior to sowing, while the remaining half was applied at jointing stage. Calcium superphosphate (150 kg P_2_O_5_ ha^-1^ year^-1^) and potassium sulphate (75 kg K_2_O ha^-1^ year^-1^) were applied as basal fertilizers.

Three soil samples at 0–20cm depth were collected from each plot using a 3cm diameter soil corer at wintering, tillering, jointing, filling and maturity stage during 2013–2014 winter wheat growing season. After mixing, the fresh samples were stored immediately in sealed plastic bags and transported to the laboratory in an insulated container. Stones, roots and other litter were removed by passing the sample through a 2mm mesh sieve and aliquots of the samples were then stored at room temperature for SOC, total N and glomalin-related soil protein analysis, and at 4°C for ammonium N, nitrate N contents and enzymatic activity analysis (within 1 week). During 2014–2015 growing season, soil samples at 20cm depth were collected only at maturity stage to measure SOC and total N.

### Soil respiration measurements

Soil respiration was measured by using a portable soil respiration system (LI-8100, Li-COR Inc., Lincoln, NE, USA) during 2013–2014 (seedling, wintering, tillering, jointing, filling and maturity stage) and 2014–2015 (seedling, tillering, jointing, heading, filling and maturity stage) winter wheat growing season. Table 1 described the specific dates of soil respiration measurement and their corresponding growth stages. One week before the first soil respiration measurement, a PVC collar (20 cm diameter, 10 cm height) was inserted 8cm into the soil surface in the middle of each plot. Living plants inside and adjacent to the PVC collar were removed regularly to avoid their effects on soil respiration. We measured soil respiration between 09:00 and 11:00 am in daytime, and at the same time soil temperature at 10 cm depth was measured by using thermocouples probe of LI-8100. Soil moisture was determined gravimetrically by oven-drying (105°C).

**Table 1:** Main growth stages of winter wheat during 2013–2014 and 2014–2015.

Growing season	Date	Growth stages
Winter wheat	13 October 2013	Sow
2013–2014	30 October 2013	Seedling
	26 November 2013	Wintering
	19 March 2014	Tillering
	19 April 2014	Jointing
	19 May 2014	Filling
	11 June 2014	Maturity
Winter wheat	10 October 2014	Sow
2014–2015	26 October 2014	Seedling
	21 March 2015	Tillering
	13 April 2015	Jointing
	5 May 2015	Heading
	27 May 2015	Filling
	14 June 2015	Maturity

### Chemical parameters

SOC and soil total N were measured by using a CHN element analyzer (Elementar Analysensysteme GmbH, Hanau, Germany), inorganic carbon was removed by 1 mol/L HCL before measuring SOC. NH_4_^+^ and NO_3_^-^ were extracted from soil with 1M KCl solution (1:5,w/v) for 60min to measure ammonium N (NH_4_^+^-N) and nitrate N (NO_3_^-^-N) contents using a flow injection autoanalyzer (Seal Analytical GmbH, Noderstedt, Germany).

### Biochemical parameters

Glomalin in soil was quantified as glomalin-related soil protein (GRSP). Soil sample was autoclaved for 30 min, in 20 mM citric acid at pH 7.0. After extraction, the sample was centrifuged at 10,000g for 5 min and the supernatant containing glomalin was collected and stored at 4°C to determine easily extractable glomalin-related soil protein (EEG). EEG in the extract was measured by Bradford assay, using bovine serum albumin as a standard. The activities of β-glucosidase (BG), Cellobiohydrolase (CB) and N-acetyl-β-D-glucosaminidase (NAG) were detected through Enzyme-labelled meter method expressed by DeForest (2009) [35].

### Statistical analysis

Soil respiration and temperature were fit based on the following exponential equation:

$$\text{Rs}={\text{ae}}^{\beta \text{T}}$$ (1)

where Rs is soil respiration rate (μmol m^-2^s^-1^), a is Rs (μmol m^-2^s^-1^) at a reference temperature of 0°C, β is the temperature reaction coefficient and T is soil temperature (°C).

The Q_10_, defined as the increment in soil respiration when soil temperature increases by 10°C, was calculated as follows:

$${\text{Q}}_{10}={\text{e}}^{10\beta }$$ (2)

All analyses were performed using SAS version 9.2. Two-way ANOVA was performed to examine the effect of growth stage and N addition on soil respiration. For each variable measured at different stages, the data were analyzed by one-way ANOVA using the Least Significant Difference (LSD) test (P<0.05) to make a comparison among treatments. Pearson’s correlation analyses were performed to assess the relationships between soil respiration and biochemical parameters.

## Results

### Soil moisture, temperature and respiration

**Fig 1:**
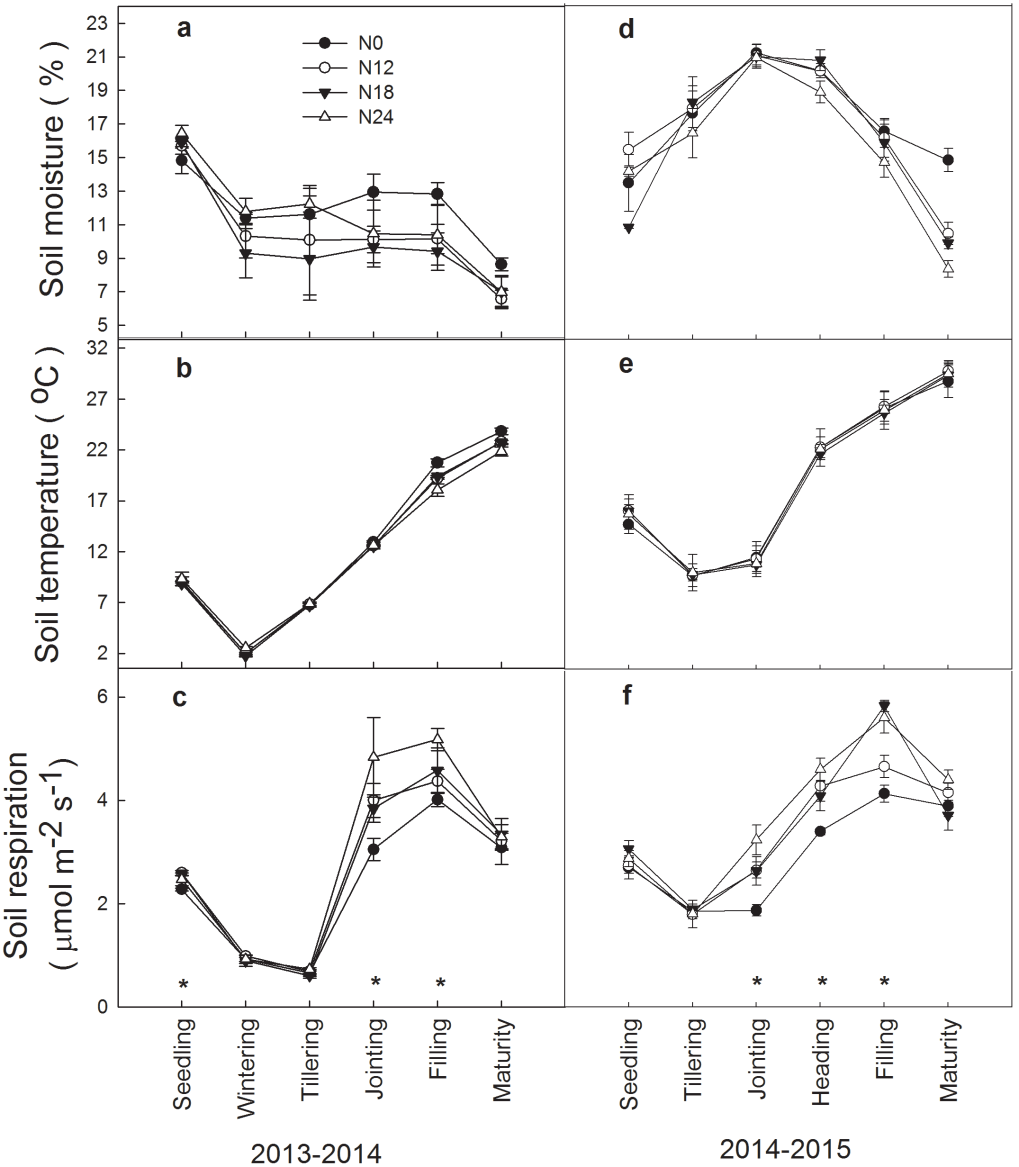
Seasonal dynamics of soil moisture, soil temperature at 10 cm depth, and soil respiration during 2013–2014 and 2014–2015 winter wheat growing seasons. Asterisks indicate significant differences between soil respiration under N0 and N24 at P<0.05.

During 2013–2014 winter wheat growing season, from seedling to wintering stage, soil moisture under all treatments decreased and was stable from wintering to filling stage, but decreased again at maturity stage (Fig 1A). However, soil moisture under all treatments showed different seasonal variation during 2014–2015 winter wheat growing season, which increased and reached maximum value at jointing stage and decreased until maturity stage (Fig 1B). Soil temperature under all treatments showed similar seasonal pattern for 2013–2014 and 2014–2015 winter wheat growing season, which decreased from seedling to wintering or tillering stage, but increased gradually until maturity stage (Fig 1C and 1D). During two consecutive winter wheat growing seasons, seasonal change of soil respiration under all treatments (Fig 1E and 1F) followed a similar pattern with soil temperature from seedling to filling stage, but decreased at maturity stage. Significant exponential relationship between soil temperature and soil respiration under different treatments was illustrated in Table 2, with Q_10_ values ranged from 2.04 to 2.46 and from 1.49 to 1.53 during two consecutive winter wheat growing season, respectively.

**Table 2:** The exponential relationship between soil respiration and temperature.

Treatments	Formulas	R^2^	P	Q_10_
Winter wheat 2013–2014				
N0	Rs = 0.8166e^0.0714T^	0.568	<0.01	2.04
N12	Rs = 0.8781e^0.0758T^	0.538	<0.01	2.13
N18	Rs = 0.7745e^0.0822T^	0.597	<0.001	2.28
N24	Rs = 0.7922e^0.0899T^	0.556	<0.01	2.46
Winter wheat 2014–2015				
N0	Rs = 1.3286e^0.0411T^	0.867	<0.001	1.51
N12	Rs = 1.5083e^0.0411T^	0.811	<0.001	1.51
N18	Rs = 1.5949e^0.0397T^	0.660	<0.001	1.49
N24	Rs = 1.5975e^0.0424T^	0.681	<0.001	1.53

Rs: Soil respiration; T: Soil temperature at 10 cm depth. The Q_10_ value was obtained from β, Q_10_ = e^10β^.

**Fig 2:**
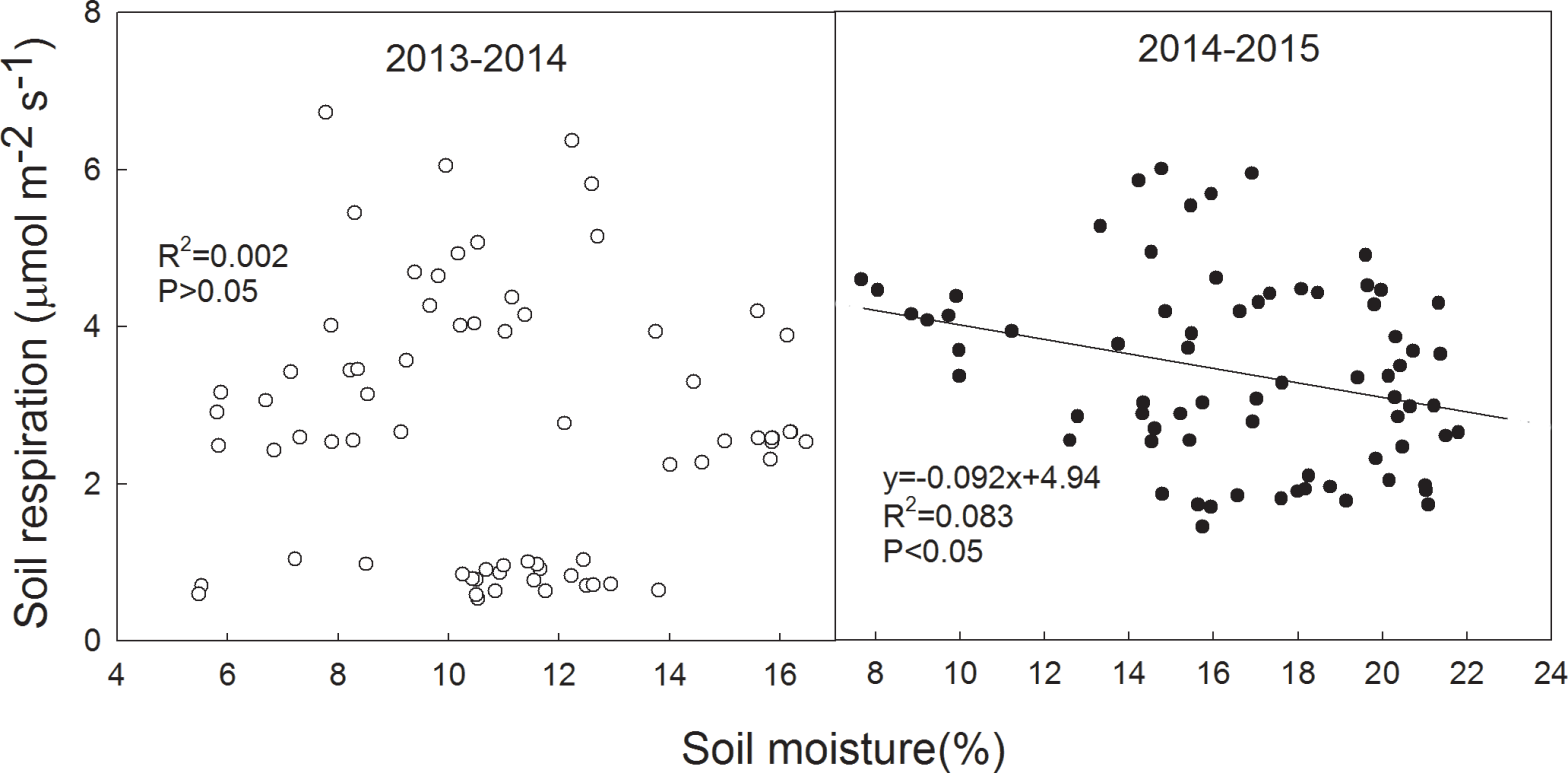
Relationship between soil respiration and soil moisture at 10 cm depth under all treatments during 2013–2014 and 2014–2015 winter wheat growing seasons.

Two-way ANOVA showed that soil respiration increased significantly with the N addition (Table 3). During 2013–2014 winter wheat growing season, compared with N0, N24 significantly increased soil respiration by 9%, 58% and 29% at seedling, jointing and filling stage, respectively (Fig 1E). The significant differences at p<0.05 level between N0 and N24 were also found at jointing, heading and filling stage during 2014–2015 winter wheat growing season (Fig 1F). Soil respiration had no significant correlation with soil moisture during 2013–2014 winter wheat growing season, whereas it was significantly and negatively correlated with soil moisture during 2014–2015 winter wheat growing season (Fig 2).

**Table 3:** Results of two-way ANOVA on the effect of growth stage and N addition on soil respiration during 2013–2014 and 2014–2015 winter wheat growing season.

	F	P value
Growing season	Stage N Stage×N	Stage N Stage×N
2013–2014	327.49 10.8 3.19	<0.0001 <0.0001 0.001
2014–2015	266.86 30.65 6.49	<0.0001 <0.0001 <0.0001

### Soil biochemical properties

**Fig 3:**
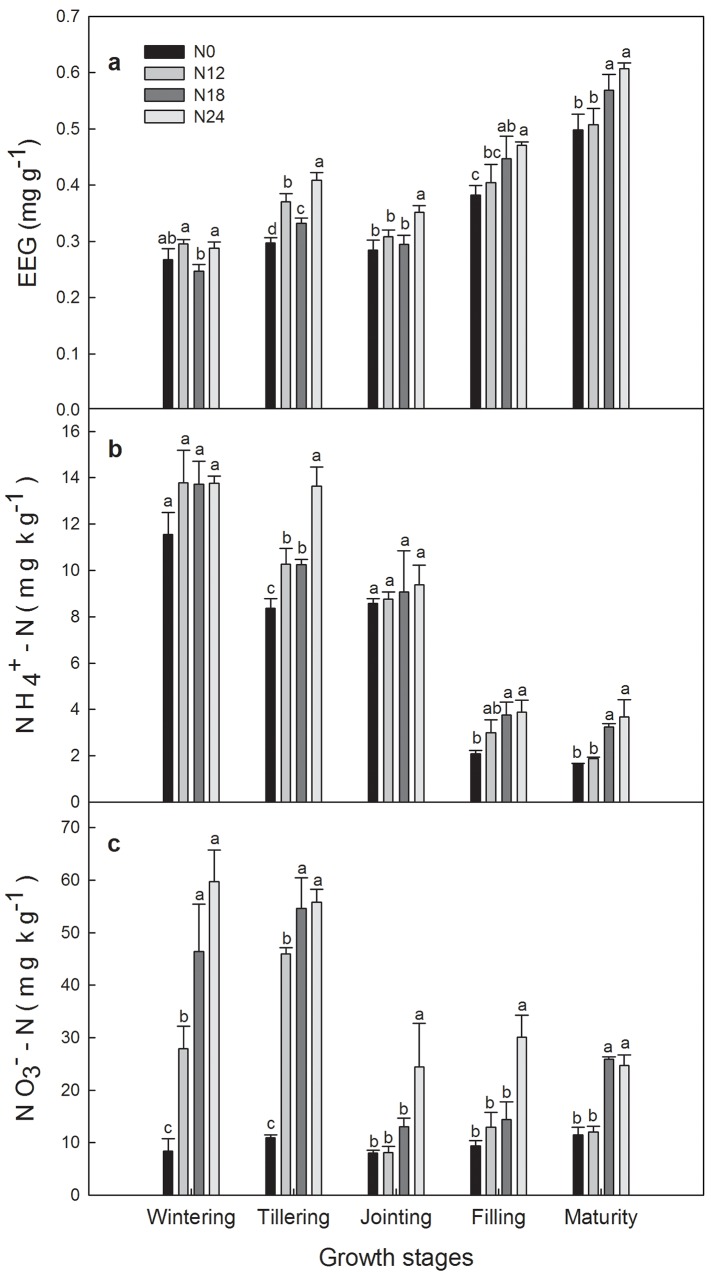
Seasonal dynamics of EEG, ammonium N (NH_4_^+^-N) and nitrate N (NO_3_^-^-N) contents among N treatments during 2013–2014 winter wheat growing season. Significant differences (P<0.05) among N treatments are indicated by different letters.

EEG content ranged from 0.25 mg g^-1^ to 0.61 mg g^-1^ and increased significantly in response to N addition (i.e. N24>N0) during the whole growing season except wintering and tillering stage (Fig 3A). EEG content showed significant seasonal variation, which increased from wintering to tillering stage, decreased at jointing stage and increased again until maturity stage.

Soil NH_4_^+^-N concentration under all treatments was maximal at wintering stage and decreased with following growth stages (Fig 3B). Significant difference (p<0.05) between N0 and N24 was observed at tillering, filling and maturity stage, soil NH_4_^+^-N concentration under N24 was at the highest level during the whole growing season. Soil NO_3_^-^-N concentration decreased markedly from tillering to jointing stage and was stable until to maturity stage (Fig 3C). Soil NO_3_^-^-N concentration under N24 was significantly higher than N0 during the whole growing season. SOC ranged from 8.61 to 9.47 mg g^-1^ and from 8.94 to 9.60 mg g^-1^ at maturity stage in 2014 and 2015, respectively. In both 2014 and 2015, SOC under N0 was significantly lower compared with N12, N18 and N24. Soil total N content ranged from 0.93 to 1.02 g kg^-1^ and 0.92 to 1.06 g kg^-1^ in 2014 and 2015, respectively (Table 4). N18 had the highest soil total N content among treatments, and increased it by 9.7% and 14.0% compared with N0 in 2014 and 2015, respectively.

**Table 4:** SOC and total N content among various N treatments at maturity stage.

Treatments	SOC (g kg^-1^)		Total N (g kg^-1^)	
	Winter wheat 2013–2014	Winter wheat 2014–2015	Winter wheat 2013–2014	Winter wheat 2014–2015
N0	8.61±0.36b	8.94±0.13b	0.93±0.03b	0.93±0.02b
N12	9.44±0.04a	9.39±0.05a	0.93±0.03b	0.92±0.02b
N18	9.44±0.19a	9.50±0.04a	1.02±0.05a	1.06±0.02a
N24	9.47±0.18a	9.60±0.20a	1.00±0.02ab	0.98±0.04b

Significant differences (P<0.05) among N treatments are indicated by different letters.

### Enzymatic activities

**Fig 4:**
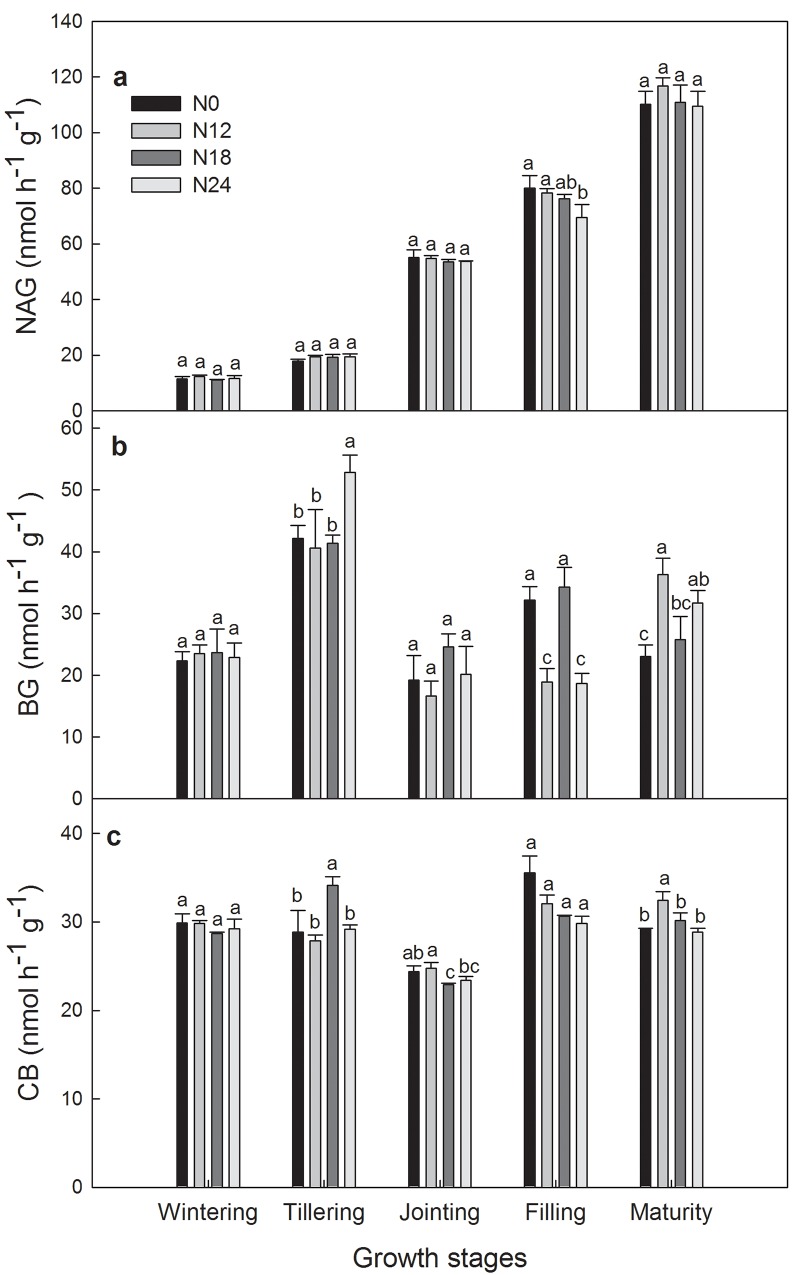
Changes of soil enzymatic activities among different N treatments during 2013–2014 winter wheat growing season. Figures followed by the same letter are not significantly (P<0.05) different, according to LSD test. NAG: N-acetyl-β-D-glucosaminidase activity; BG: β-glucosidase activity; CB: Cellobiohydrolase activity.

NAG activity was not significantly affected by N addition except filling stage where NAG activity under N24 was significantly lower than N0 and N12. From wintering to tillering stage, NAG activity was constant under all treatments, and then increased to reach their maximal values at maturity stage (Fig 4A). BG activity under all treatments showed similar seasonal changing trends, which increased from wintering to tillering stage, decreased to the lowest value at jointing stage, and increased again until maturity stage except N0 and N18 which showed contrary trends from filling to maturity stage (Fig 4B). BG activity under N24 was significantly higher than N0 at tillering and maturity stage. CB activity under N12 and N24 was stable at the first two stages, reached the lowest value at jointing stage, increased at filling stage, and became stable during last two stages (Fig 4C).

### Correlation between soil respiration and biochemical properties

Soil respiration was significantly and positively correlated with BG activity (p<0.05), EEG (p<0.001), SOC (p<0.001), total N (p<0.01), ammonium N (p<0.001) and nitrate N contents (p<0.001) (Table 5). BG activity also showed a significant positive correlation at p<0.05 level with EEG, total N, NH_4_^+^-N and NO_3_^-^-N concentrations. In addition, EEG content was significantly and positively correlated with SOC (p<0.01), total N (p<0.001), ammonium N (p<0.001) and nitrate N contents (p<0.001).

**Table 5:** Correlation matrix of soil respiration and soil biochemical parameters by using winter wheat growing season means during 2013–2014.

	Rs	NAG	BG	CB	EEG	NH_4_^+^-N	NO_3_^-^-N	SOC
NAG	NS							
BG	0.562*	NS						
CB	NS	0.603*	NS					
EEG	0.874***	NS	0.619*	NS				
NH_4_^+^-N	0.878***	NS	0.554*	NS	0.947***			
NO_3_^-^-N	0.889***	NS	0.636*	NS	0.850***	0.903***		
SOC	0.828***	NS	NS	NS	0.758**	0.865***	0.764***	
TN	0.641**	NS	0.630*	NS	0.844***	0.841***	0.730**	0.661**

Rs: Soil respiration; NAG: N-acetyl-β-D-glucosaminidase activity; BG: β-glucosidase activity; CB: Cellobiohydrolase activity; EEG: Easily extractable BRSPs; NH_4_^+^-N: Ammonium N content; NO_3_^-^-N: Nitrate N content; SOC: Soil organic carbon; TN: Soil total N.

NS: Not significant

* P<0.05

** P<0.01

*** P<0.001

## Discussion

### Seasonal variation of soil respiration, temperature and moisture

Pronounced seasonal variations of soil respiration during winter wheat growing season were also reported by many previous studies [36–37]. Seasonal changes in soil respiration are generally attributed to the changes in soil temperature [12, 38–40]. Falling soil temperature led to a decrease of soil respiration between seedling and tillering stage, which corresponded with Zhang et al. (2013) [17]. A maximum soil respiration value appeared at filling stage because microbial and root activities were high at optimum soil temperature [41], whereas the senescence of roots resulted in significant decline of soil respiration at maturity stage.

A significant exponential relationship between soil respiration and temperature was found in our results, which was consistent with previous studies [42–43]. Some researchers used Q_10_ value to indicate a strong relationship between soil respiration and temperature [44–45]. The range of Q_10_ in our study agreed with the findings of many previous results [21, 46]. Soil temperature explained about 66% of soil respiration (R^2^ average, Table 2), so it indicated that Q_10_ is a good indicator to evaluate the sensitivity of soil respiration to soil temperature during winter wheat growing season of our study. Gong et al. (2014) reported that soil moisture had a significant and negative effect on Q_10_ when soil moisture was less than 50% [21]. In our study, soil moisture of 2014–2015 winter wheat growing season was higher than that of 2013–2014. So the increase of soil moisture resulted in the decrease of Q_10_.

Soil moisture was another important factor to alter soil respiration [19, 47]. Comparing with no significant correlation between soil respiration and moisture during 2013–2014 winter wheat growing season, a significant and negative correlation between soil respiration and moisture during 2014–2015 winter wheat growing season was found in our study, which might be due to poor gas diffusion in surface soils and reduction in activity of obligate aerobic microbes caused by excessive water [8].

### Response of soil respiration to N addition

There are contradictory viewpoints about whether the effects of N fertilization on soil respiration are positive [10, 48] or negative [9, 49]. In general, effects of N addition on soil respiration varied with time (in terms of growth stages) according to our results. No significant effect of N addition on soil respiration was found during 2013–2014 (wintering and tillering stage) and 2014–2015 winter wheat growing season (tillering stage), because soil temperature was low (1–7°C) at the two stages (Fig 1E and 1F), which suppressed microbe and root activities and then weakened the effect of N addition on soil respiration. Soil respiration under N24 was higher than N0 at optimum soil temperature (i.e. jointing, heading and filling stage), which agreed with previous results [10, 48]. This implied that effect of N addition on microbe and root activities tends to intensify at optimum soil temperature, and then a significant difference in soil respiration between N0 and N24 appeared. Soil respiration is composed of both autotrophic respiration (Ra) and heterotrophic respiration (Rh) [39, 50]. The effect of root senescence on Ra masked effect of N addition on soil respiration, this might partly explain the finding that there was no significant effect of N addition on soil respiration at maturity stage.

### Relationship between soil respiration and biochemical parameters

Microbes would produce more enzymes if available nutrients were scarce [51–52]. Therefore, abundant freely available mineral N in the soil did not stimulate microbes to produce more NAG, and then resulted in no significant correlation between NAG activity and NH_4_^+^-N and NO_3_^-^-N concentrations. As a component part of available N, NH_4_^+^-N and NO_3_^-^-N tend to encourage more root growth [53]. Some studies suggested that root biomass significantly positively correlated with soil respiration [21, 37]. Significant and positive correlation between BG activity and NH_4_^+^-N and NO_3_^-^-N contents also indicated that NH_4_^+^-N and NO_3_^-^-N increased Rh by stimulating soil enzyme activity, and then enhanced soil respiration. These might be the reason why significant positive correlations between soil respiration and NH_4_^+^-N and NO_3_^-^-N concentrations was observed in our study.

EEG showed a significant positive correlation with BG activity in the current study. This might be explained as follows, enzymatic activities were used as indicators of microbe activity because of altering biochemical processes [54]. As an important microbe, arbuscular mycorrhizal fungi (AMF) might produce more glomalin when enzymatic activities were high. The SOC pool, an important component of terrestrial ecosystems, is a crucial regulator of carbon fluxes between biosphere and atmosphere [23]. Significant and positive correlation between EEG and SOC in our study confirmed that positive effect of glomalin on organic carbon preservation [55]. NH_4_^+^-N and NO_3_^-^-N concentrations were significantly and positively correlated with EEG, consisting with previous studies [23, 56].

Applying nitrogen fertilizer decreases soil C:N and increases microbial demand for C [57], so microbes synthesize enzymes that involved in the breakdown of soil organic matter to meet this demand. So a positive and significant correlation between BG activity and NH_4_^+^-N and NO_3_^-^-N contents was observed. BG decomposed cellobiose to glucose (a major C source for microbes) and then resulted in significant positive correlation between BG activity and soil respiration, which agreed with previous studies [25, 58].

## Conclusions

Soil respiration is manipulated by not only abiotic factors (soil moisture, temperature and nitrogen addition) but also by biotic factors (soil enzyme activity and glomalin content). Therefore, soil enzyme activity and glomalin content can be useful indicators for soil carbon management in cropland ecosystem. N addition stimulates cellulose-degrading enzyme (BG) activity but has no significant influence on chitinase-degrading enzyme (NAG) activity in this specific soil type and climate condition. 180 kg N ha^-1^ year^-1^ (N18 treatment) is recommended for local farmer practice because it not only leads to a decrease of C loss but also improves soil biochemical properties. The significant positive effect of 3 years N addition on soil respiration is found in our study and further research is needed to determine the impact of chronic N application on soil respiration. In addition, quantifying components of soil respiration in future research is critical to fully understand mechanism that regulates the effect of N addition on soil respiration.

## Supporting Information

S1 Dataset S1 Dataset is the data of soil respiration, moisture, temperature and biochemical properties. This data contain two parts: 1. Soil respiration, moisture and temperature under N0, N12, N18 and N24 treatment during 2013–2014 and 2014–2015 winter wheat growing seasons. 2. Soil biochemical properties under N0, N12, N18 and N24 treatment during 2013–2014 and 2014–2015 winter wheat growing seasons.
(XLSX)
